# Influence of Root Canal Treatment on Oral-Health-Related Quality of Life (OHRQoL) in Saudi Patients: A Cross-Sectional Study

**DOI:** 10.7759/cureus.45035

**Published:** 2023-09-11

**Authors:** Montaha Alsultan, Swati Srivastava, Muhammad Qasim Javed, Mansoor Khan, Hamza Ulfat

**Affiliations:** 1 Department of Conservative Dentistry, College of Dentistry, Qassim University, Buraidah, SAU; 2 Department of Conservative Dental Sciences, College of Dentistry, Qassim University, Buraidah, SAU; 3 Department of Operative Dentistry, Foundation University College of Dentistry, Foundation University, Rawalpindi, PAK; 4 Department of Operative Dentistry, Heavy Industries Taxila Education City-Institute of Medical Sciences (HITEC-IMS) Dental College, Taxila, PAK

**Keywords:** oral-health-related quality of life, root canal treatment, oral health impact profile, oral health, endodontic therapy

## Abstract

Objective

This cross-sectional study aimed to assess the influence of root canal treatment on the oral-health-related quality of life (OHRQoL) of patients in the Kingdom of Saudi Arabia (KSA). The data on KSA’s population are significantly limited, highlighting the significance of additional research to be carried out in this particular field.

Methods

The study was conducted at the dental clinics of the College of Dentistry, Qassim University, from January to June 2022. A total of 112 patients who had undergone endodontic therapy (ET) for teeth with irreversible pulpitis were included. Demographic data and treatment procedures were recorded. The patients' responses to the OHIP-14 questionnaire were analyzed to assess their OHRQoL. The scores were converted into qualitative categories (good, moderate, poor) for classification.

Results

The majority of patients (59.8%) reported a good OHRQoL after ET. Physical pain was the only variable where more than 50% of patients reported experiencing pain. Older age and smoking status were significantly associated with a poorer OHRQoL. However, no significant gender differences in OHRQoL were observed.

Conclusion

ET had a positive impact on the OHRQoL of patients in Saudi Arabia. The study highlights the importance of considering patient-centered outcomes, such as OHRQoL, in assessing the success of root canal treatment. Further research with longitudinal designs and randomized controlled trials is needed to better understand the long-term effects of root canal treatment on patients' OHRQoL and to compare them with other dental treatments.

## Introduction

The main purpose of endodontic therapy (ET) is to maintain or restore the health of periapical tissue (PT) by preventing or eradicating root canal space infection. Traditionally, the studies have assessed outcomes by radiographic/clinical evaluation of PT status and survival of endodontically treated teeth. Nevertheless, other outcomes related to health also have significance. Such outcomes include the effect of therapy on patients’ satisfaction and their daily life. There is a dearth of evidence-based literature on the aforementioned post-ET patient-centered outcomes [1-6].

Oral health (OH) is regarded as the basic component of general health. As a result, OH contributes to health-related quality of life (HRQOL) both at psycho-social and biological levels [7-10]. In the KSA, several authors have endeavored to determine the oral-health-related quality of life (OHRQoL) in various contexts. Oral diseases like dental caries [11], malocclusion [12,13], periodontal disease [14], and fixed dental prosthesis have been evaluated. However, the impact of root canal treatment (RCT) on OHRQoL is missing in the literature.

The oral health impact profile (OHIP) is an accepted instrument for measuring the OHRQoL. The OHIP questionnaire was originally developed as 49 questions that captured seven dimensions [15]. Subsequently, a shorter version was developed (OHIP-14) by Slade in 2006 which is composed of 14 questions and captures the dimensions of the original OHIP version [16]. OHIP-14 has been translated and validated in the Arabic Language [17]. Liu et al. conducted a case-control study where OHRQoL was assessed using the short-form Oral Health Impact Profile measure (OHIP-14) [1]. They identified significant differences in OHRQoL and psychological well-being among patients requiring endodontic treatment compared to patients in periodontal maintenance. In another longitudinal study, improvement in OHRQoL was recorded after endodontic treatment [2]. The OHIP-14 instrument was found to be both sensitive and responsive to endodontic treatment and aided in understanding patients' perspectives of outcomes from endodontic care. In a study by Liu et al., using the OHIP-14 instrument, various endodontic factors were found to be associated with OHRQoL controlling for other OH factors like dental caries, periodontal status, and socio-demographic factors [3].

While there is an abundance of research available in other nations, the available data concerning the population of Saudi Arabia are quite restricted, emphasizing the necessity for further research to be conducted in this specific field. Considering this, the current study aimed to assess the impact of ET on the patient’s ‘OHRQoL’ by using the ‘OHIP-14’ Arabic version.

## Materials and methods

Study design, setting, and sample size

This analytical cross-sectional research was conducted at the dental clinics of the College of Dentistry, Qassim University from January to June 2022. This study was ethically approved by the institutional review board of Qassim University with the Registration number EA/6133/2021, dated 17/4/2021. Using the results of a prior investigation, a sample size calculation was carried out [18]. With an effect size of 0.50, 0.05 alpha error, and 95% power, a minimum sample of 80 participants was calculated as the required sample size. The convenience sampling technique was used. The study included patients who underwent ET in teeth with irreversible pulpitis. Pregnant patients and patients with incomplete ET, a history of chronic pain medication use, cognitive issues, and uncontrolled systemic disorders were excluded. Patients returning at least one week after endodontic treatment were enrolled in the study. Out of 121 patients who were approached to participate in the study, 112 patients agreed to fill out the study questionnaire. The patients were provided with the study information and the consent forms. Informed consent was obtained from all the participants who agreed to participate. The data for the children were collected with the assistance and consent of their parents.

All of the enrolled patients were treated by endodontic specialists using rotary NiTi instruments (ProTaper, Dentsply Malliefer, Switzerland). The majority (78.2%) of these treatments were completed in a single visit and 21.8% were performed in multiple visits. The canals were disinfected using sodium hypochlorite (ChlorCid, Ultradent, USA). Calcium hydroxide was used as intracanal medicament in teeth that were treated in multiple visits (UltraCal XS, Ultradent, USA). The teeth that were tender to percussion and had at least one weeping canal were treated in multiple visits. All of the teeth were obturated with ProTaper matching gutta percha points (Protaper, Dentsply Malliefer, Switzerland) using the single cone technique and bioceramic sealer (Bio-C Sealer - Angelus Odontologia). The access cavities were restored using composite resin. 

Data collection and instrument

The OHIP (Arabic version) was utilized for data collection [17]. The validity and reliability of OHIP-Arabic have been established in the previous study [13], with a Kappa value of 0.89 and an OHIP subscale-OHIP total score correlation range between 0.64 and 0.77 [13]. OHIP-14 is a self-administered questionnaire that is composed of 14 questions on a five-point Likert scale: 0 (very often); 1 (fairly often); 2 (occasionally); 3 (hardly ever); 4 (never). OHIP-14 assesses quality of life by collecting responses across seven dimensions (two questions each per dimension) as follows: (i) functional limitation; (ii) psychological discomfort; (iii) physical pain; (iv) physical disability; (v) social disability; (vi) psychological disability, and (vii) handicap. The previous research studies have utilized OHIP-14 with endodontic discipline. The results of the studies have suggested that pulpal pathosis might influence the patient’s perceived OHRQOL [1-3]. Considering this, OHIP-14 was used in the current study.

The question text for one question was changed from “How often you had problems with your teeth, mouth and dentures” to “How often you had problems with your teeth and gums” to maintain focus on effects of endodontic treatment on the teeth, pulp and periapical tissues.

The score from the OHIP questionnaire was converted into qualitative categories (good, moderate, poor) [19]. A score of 0 was allocated to the response (very often) on one end of the Likert scale and a score of 4 was allocated to the response (never) on the other end. The total score for each patient was then calculated by adding the score obtained from the response of each question. This summation was then divided by 56 (the maximal possible score) and multiplied by 100 to obtain a percentage of the patients' scores. This was done for simplification of data interpretation. Patients who scored more than 65% were designated as having good OHRQoL. Patients scoring between 50 and 65% were designated as having moderate OHRQoL and patients having a score less than 50% were considered as having a poor OHRQoL.

Data analysis was done by using IBM SPSS Statistics for Windows, Version 24 (Released 2016; IBM Corp., Armonk, New York, United States). Descriptive statistics were documented as frequency and percentage descriptions. The categorical variables were compared by utilizing the chi-square test. The level of significance was set at a p-value of <0.05.

## Results

The 112 patients who participated in the research gave precise and comprehensive responses. The mean age of the respondents was 30.7±12.62 years, ranging from 10 to 66 years. The respondents were divided into three groups according to the age from 21-40 years (53.7%), less than 20 years (24%), and 41-60 years (22.3%). More than half of the respondents were females (51.2%). The majority of respondents were single (60.3%) and non-smokers (89.3%). Out of 112 study participants, 72.3% underwent endodontic treatment more than two weeks ago (Table 1).

**Table 1:** Distribution of respondents in terms of the basic demographic data and history.

Variables	N (%)
Gender:	
Male	59 (48.8)
Female	62 (51.2)
Age group (in years):	
<20	29 (24)
21-40	65 (53.7)
41-60	27 (22.3)
Social status:	
Single	73 (60.3)
Married	48 (39.7)
Educational level:	
Nothing	5 (4.13)
Basic education	11 (9.09)
High school education	41 (33.88)
High education	18 (14.88)
University education	46 (38.02)
Smoking:	
Yes	13 (10.7)
No	108 (89.3)
Do you suffer from any health problems?	
Yes	9 (7.4)
No	112 (92.6)
Have you had root canal treatment?	
No	9 (7.4)
Yes	112 (92.6)
How long ago you had root canal treatment?	
One to two weeks	31 (27.7)
More than two weeks	81 (72.3)

Table 2 depicts the item and dimension-wise distribution of the participants’ responses who underwent ET (n=112) on OHIP-14. The majority of patients (75.9%) had never faced an obstacle in pronouncing the words or felt a worsened taste (67%) because of oral problems. However, 40.2% and 45.5% occasionally had painful aching in the mouth or felt uncomfortable to eat some foods, respectively. About 62.5% had never felt insecure because of their oral problems while 30.4% had occasionally felt tense because of their mouth problems. Most of the respondents (61.6%) had never felt the diet was unsatisfactory because of teeth or mouth problems. Only 34.8% had occasionally been interrupted while eating meals due to oral pain. Also, 33.9% had never found it difficult to relax while 22.3% had occasionally felt difficulty relaxing. As for being embarrassed or irritable, 41.1% and 39.3% had never had a problem because of their oral diseases. According to most of the patients, they have never or hardly ever had felt difficulty doing their usual job, unsatisfied with their life, or being totally unable to function.

**Table 2:** Dimension-wise distribution responses of the participants who underwent endodontic therapy (n=112) on OHIP-14.

Oral Health Impact Profile-14 with 7- subdomains (Questionnaire)	How often have you had problem in the last year				
	Very often	Fairly often	Occasionally	Hardly ever	Never
	N (%)	N (%)	N (%)	N (%)	N (%)
Functional Limitation					
Have you had trouble pronouncing any words because of problems with your teeth and gums?	4 (3.6)	4 (3.6)	7 (6.3)	12 (10.7)	85 (75.9)
Have you felt that your sense of taste has worsened because of problems with your teeth and gums?	2 (1.8)	4 (3.6)	19 (17)	12 (10.7)	75 (67)
Physical Pain					
Have you had painful aching in your mouth?	6 (5.4)	13 (11.6)	45 (40.2)	21 (18.8)	27 (24.1)
Have you found it uncomfortable to eat any foods because of problems with your teeth, mouth or dentures?	15 (13.4)	12 (10.7)	51 (45.5)	20 (17.9)	14 (12.5)
Psychological Discomfort					
Have you been self-conscious because of your teeth, mouth or dentures?	3 (2.7)	2 (1.8)	15 (13.4)	22 (19.6)	70 (62.5)
Have you felt tense because of problems with your teeth, mouth or dentures?	12 (10.7)	8 (7.1)	34 (30.4)	26 (23.2)	32 (28.6)
Physical Disability					
Has your diet been unsatisfactory because of problems with your teeth, mouth or dentures?	5 (4.5)	5 (4.5)	21 (18.8)	12 (10.7)	69 (61.6)
Have you had to interrupt meals because of problems with your teeth, mouth or dentures?	5 (4.5)	5 (4.5)	39 (34.8)	30 (26.8)	33 (29.5)
Psychological Disability					
Have you found it difficult to relax because of problems with your teeth, mouth or dentures?	14 (12.5)	12 (10.7)	25 (22.3)	23 (20.5)	38 (33.9)
Have you been a bit embarrassed because of problems with your teeth, mouth or dentures?	12 (10.7)	6 (5.4)	26 (23.2)	22 (19.6)	46 (41.1)
Social Disability					
Have you been a bit irritable with other people because of problems with your teeth, mouth or dentures?	5 (4.5)	4 (3.6)	30 (26.8)	29 (25.9)	44 )39.3)
Have you had difficulty doing your usual jobs because of problems with your teeth, mouth or dentures?	5 (4.5)	4 (3.6)	18 (16.1)	34 (30.4)	51 (45.5)
Handicap					
Have you felt that life in general was less satisfying because of problems with your teeth, mouth or dentures?	6 (5.4)	7 (6.3)	30 (26.8)	34 (30.4)	35 (31.3)
Have you been totally unable to function because of problems with your teeth, mouth or dentures?	4 (3.6)	12 (10.7)	24 (21.4)	20 (17.9)	52 (46.4)

 More than half of the patients (59.8%) had good quality of life followed by 33% having a moderate OHRQoL (Table 3).

**Table 3:** Distribution of the respondents who underwent endodontic therapy (n=112) regarding OHRQoL. OHRQoL: Oral-health-related quality of life

Oral-Health-Related Quality of Life	N (%)
Good	67 (59.8)
Moderate	37 (33)
Low	8 (7.1)
Total	112 (100)

The relation between the total OHRQoL score and the demographics of the studied patients showed that the older age and smoking status were significantly associated with a poor OHRQoL (Table 4).

**Table 4:** Relation between the total scores based on OHIP-14 and demographic data. *Statistically significant; N.S.: non-significant; OHRQoL: oral-health-related quality of life

Variable	OHRQoL						p-value
	Good		Moderate		Low		
Age							
Range	10.0-66.0		12-56.0		23-56.0		0.014*
Mean + SD	29.14+12.62		31.08+11.07		42.75+14.24		
Sex	N (%)		N (%)		N (%		
Male	30 (44.8)		17 (45.9)		6 (75)		0.264 N.S.
Female	37 (55.2)		20 (55.1)		2(25)		
Smoking	N (%)		N (%)		N (%)		
No	64(95.5)		31 (83.8)		5 (62.5)		0.007*
Yes	3 (4.5)		6 (16.2)		3 (37.5)		
Comorbidity	N (%)		N (%)		N (%		
No	64(95.5)		32 (86.5)		7 (87.5)		0.239 N.S.
Yes	3 (4.5)		5 (13.5)		1 (12.5)		
Duration of root canal treatment	OHRQoL						p-value
	Good		Moderate		Low		
One to two weeks	11	16.4	15	40.5	5	62.5	0.002*
More than two weeks	56	83.6	22	59.5	3	37.5	

## Discussion

RCT is a widely used endodontic procedure aimed at preserving tooth functionality while reducing discomfort and pain. Our findings indicate that a significant majority (n=67, 59.8%) of patients reported a good OHRQoL after undergoing ET. Notably, physical pain was the only variable where the participants scored more than 50% in very often, fairly often, and occasionally categories combined. Forty-eight patients (42.9%) experienced mild or no pain after the procedure while 64 (57.2%) reported some form of pain. Pain is an important factor that influences patient satisfaction and overall OHRQoL. Vena et al. reported OHRQoL to be significantly impacted (p < 0.0001) by persistent pain after RCT [20].

All the RCTs conducted in our study were completed in a single visit. The literature shows that the type of treatment protocol is also reported to affect the OHRQoL after the RCT. A study by Ezzat et al. reported an improvement in the OHIP-14 score by 7.6 points (p = 0.001) in the single-visit RCT group and by eight points (p = 0.001) in the multiple-visit RCT group [21].

The patients in the present study were treated using the rotary system. A study by Pasqualini et al. comparing reciprocating and rotary systems found that those treated with the reciprocating system experienced a slower reduction in pain, faced greater challenges with eating, sleeping, and performing daily activities, and encountered more difficulties in their social interactions compared to the group treated with a rotary system [22]. One possible reason for these results could be attributed to the higher expulsion of debris when using the reciprocating system [23]. However, in a study comparing two different root canal treatment protocols (Protaper Next and Reciproc), Oliveira et al. found no significant difference in OHRQoL after RCT using these systems [24].

Regarding age, our study revealed that older patients tended to have a poorer OHRQoL. Interestingly, this contrasts with the findings of Zilinskaite-Petrauskiene and Haug where elderly patients surprisingly reported significantly better (p < 0.05) OHRQoL (OHIP-14 score = 4.93 ± 6.74) compared to younger individuals (OHIP score = 7.41 ± 7.88) [25]. Similarly, a recent study by Johnsen et al. also reported a significantly better OHIP-14 score (P<0.1) in the elderly (3.02 ± 4.45) compared to younger patients (7.47 ± 8.63) [26]. This difference may be attributed to older individuals generally perceiving oral health as having a reduced impact on their overall quality of life compared to younger counterparts, as observed in their studies [27].

Furthermore, our study revealed a difference between men and women in OHRQoL. Women reported a better OHRQoL in our study. However, the results were not significant. A research study by Zilinskaite-Petrauskiene and Haug indicated that women requiring RCT reported a significantly lower quality of life (p < 0.01) compared to men (8.20 ± 8.81 and 4.44 ± 5.44, respectively [25]. This observation of women experiencing poorer oral health-related quality of life has also been noted in other Scandinavian epidemiological studies; Johnsen et al. reported a poor OHIP-14 score in women (7.14 ± 9.44) compared to men (4.90 ± 5.64) [26]. However, the results were not significant. Possible explanations for this gender difference could be women's greater concern for appearance and health, leading to a more significant impact of endodontic problems on their quality of life. Additionally, previous research by Caltabiano et al. showed that women tend to exhibit more anxiety before receiving treatment compared to men (Modified Dental Anxiety Score 11.93 compared to 9.94 respectively) [28]. However, it is important to consider that the responses in our study were recorded after the RCT, potentially accounting for contrasting results from previous studies.

The questionnaire was administered to patients who had undergone RCT 1 to 2 weeks before, acknowledging that OHIP scores can show improvement of 40% one month and 50% six months after treatment [2]. Although Liu et al. and Wigsten et al. recommend a six-month recall to assess peri-radicular healing, noticeable clinical improvement is already evident after two weeks, as postoperative pain decreases significantly during this period [2,29].

Limitations

The limitation of this study is its cross-sectional design, which did not allow the comparison of the ‘OHRQoL’ of patients pre- and post-treatment.

## Conclusions

In conclusion, our study sheds light on the impact of endodontic therapy on patients' QOL, showing improvements in the seven dimensions of OHIP-14 with 59.8% and 33% of patients documenting good and moderate OHRQOL, respectively. Further research is needed to better understand the influence of age and gender on OHRQoL outcomes. Additionally, future research studies with longitudinal designs should be planned to assess the long-term effects of root canal treatment on patients' oral health outcomes and QOL and compare them to other dental services such as restorative, prosthodontic, and periodontal treatments.
